# Lane and Traffic Sign Detection for Autonomous Vehicles: Addressing Challenges on Indian Road Conditions

**DOI:** 10.1016/j.mex.2025.103178

**Published:** 2025-01-20

**Authors:** H. S. Gowri Yaamini, Swathi K J, Manohar N, Ajay Kumar G

**Affiliations:** a Department of Computer Science, Amrita School of Computing, Amrita Vishwa Vidyapeetham, Mysuru 570026, India; b Department of Mining and Explosive, Engineering Missouri University of Science and Technology, MO 65409-1060, USA

**Keywords:** Object detection, Transfer learning, Convolutional block attention mechanism (CBAM), Data augmentation, Ghost convolution, YOLOv8 Transfer Learning

## Abstract

Accurate and precise detection of lanes and traffic signs is predominant for the safety and efficiency of autonomous vehicles and these two significant tasks should be addressed to handle Indian traffic conditions. There are several state-of-art You Only Live Once (YOLO) models trained on benchmark datasets which fails to cater the challenges of Indian roads. To address these issues, the models need to be trained with a wide variety of Indian data samples for the autonomous vehicles to perform better in India. YOLOv8 algorithm has its challenges but gives better precision results and YOLOv8 nano variant is widely used as it is computationally less complex comparatively. Through rigorous evaluations of diverseness in the datasets, the proposed YOLOv8n transfer learning models exhibits remarkable performance with a mean Average Precision (mAP) of 90.6 % and inference speed of 117 frames per second (fps) for lane detection whereas, a notable mAP of 81.3 % for traffic sign detection model with a processing speed of 56 fps.

• YOLOv8n Transfer Learning approach by adjusting architecture for lane and traffic sign detection in Indian diverse Urban, Suburban, and Highway scenarios.
• Dataset with 22,400 images of normal and complex Indian scenarios include crude weathering of roads, traffic conditions, diverse tropical weather conditions, partially occluded and partially erased lanes, and traffic signs.
• The model performance with notable precision and frame wise inference.

## Graphical abstract

**Figure:**
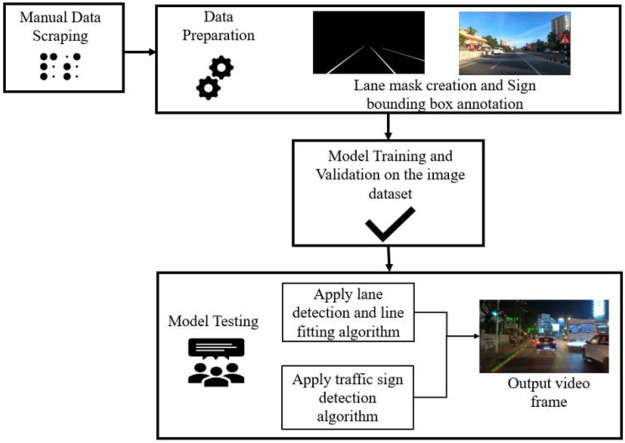

Specifications table

**Table:** 

Subject area:	Computer Science
More specific subject area:	Image and Video Processing
Name of your method:	YOLOv8 Transfer Learning
Name and reference of original method:	NA
Resource availability:	Ultralytics yolov8

## Background

Lane and traffic sign detection in deep learning involves identifying objects in images or videos, essential for applications such as self-driving vehicles, surveillance, clinical imaging, and robotics. The autonomous vehicle domain focuses on locating predefined objects like lanes, cars, pedestrians, traffic signals, and animals by identifying bounding boxes. Lane detection is critical for precise navigation, employing computer vision techniques and deep learning algorithms to identify road markings and outline vehicle paths. Similarly, traffic sign detection is essential for informed decision-making, adherence to regulations, and navigation optimization. Technologies like YOLO are crucial for evaluating detection algorithms' accuracy, speed, and robustness and this paper aims to develop perception systems capable of accurately detecting both lanes and traffic signs, particularly on Indian roads to develop intelligent systems capable of navigation in complex road scenarios. The proposed system can be extended beyond autonomous vehicles, contributing to surveillance systems, advanced computer vision in complex environments and the system's adaptability holds significant promise for adopting it on similar road conditions globally with emerging technologies.

The existing research collectively investigates diverse methodologies to advance object detection and recognition. Notable contributions from Luo et al. [1] and Soylu & Soylu [2] focus on real-time traffic sign detection, leveraging techniques like YOLOv8 to achieve remarkable accuracy and speed. The work by Zhang et al. [3] focuses on small target detection and studies by Wang et al. [4] and Liu et al. [5] tackle challenges such as dense crowds and pavement crack identification using refined iterations of the YOLOv8 model, showcasing elevated precision and recall rates. Despite prominent progress, challenges like effectively managing diverse scenarios, managing computational resource demands, and conducting robust analysis exists. Techniques such as data augmentation and lightweight architectures show promise for practical applications and continued exploration and fine-tuning are essential to support scalability and generalization.

**Fig. 1:**
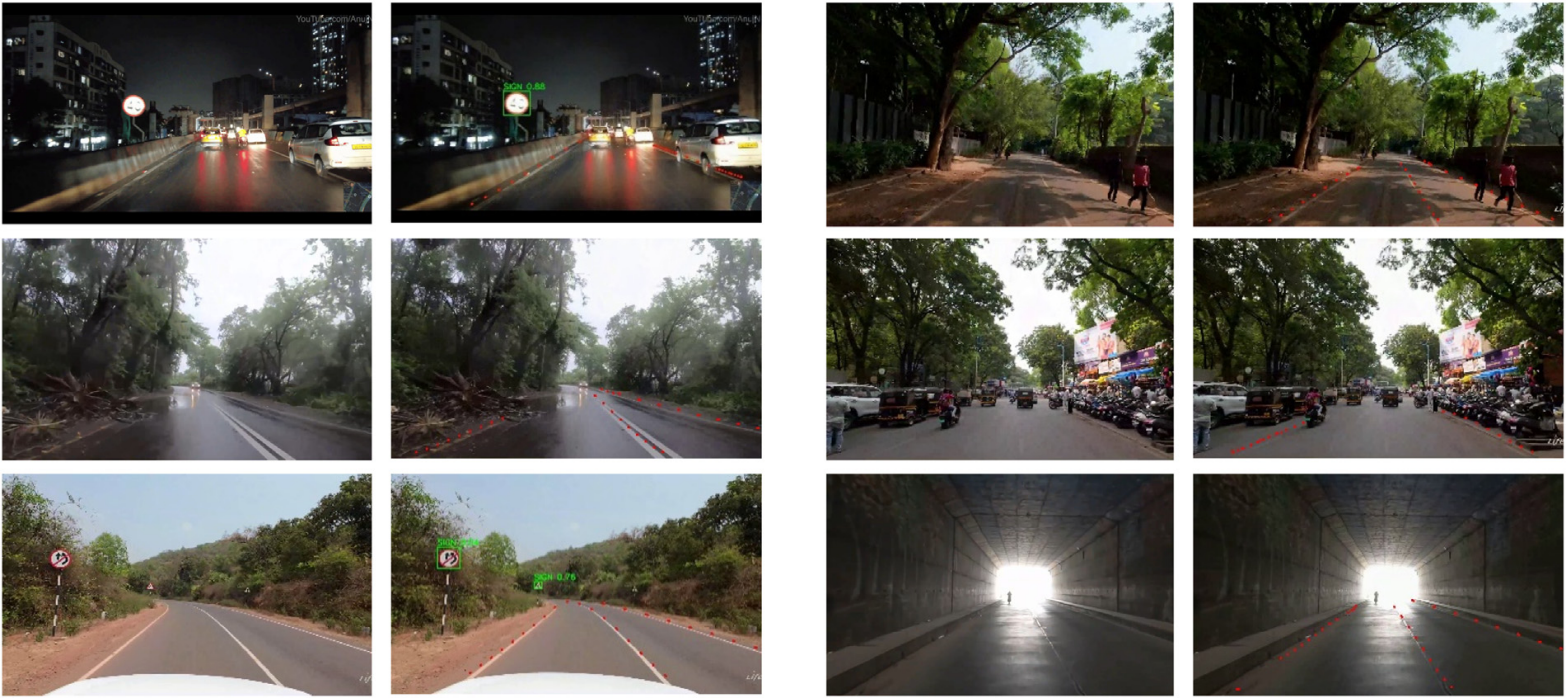
Different Indian road challenges addressed along with the results of the proposed study.

This proposed system addresses the challenges within Indian road scenarios like partially occluded or erased lanes and traffic signs, crowded roads with unsystematic traffic regulations along with pedestrians and animals on the road, roads with intricate curves, roads under varied times of day and weather conditions like rain, fog, and mist, and harsh weathering of traffic sign boards and lane markings in urban, suburban and highway instances. While current state-of-art models excel with benchmark datasets, they underperform when deployed on Indian traffic scenarios, hence, a model which is trained against such challenges is required. YOLOv8 is an advanced object detection model that is built as a successor upon the fame of previous YOLO versions such as YOLOv5, YOLOv7, etc., and introduces new features and improvements in techniques such as object detection, object tracking, instance segmentation, image classification, and pose estimation tasks. The nano variant is widely used because it maintains a good balance between speed and accuracy which is suitable for applying in studies and application with limited computational resources and is computationally less complex when compared to YOLOv8l and YOLOv8x variants. To attend such a demand, the YOLOv8n model is adjusted with additional convolutional layers for lane segmentation and traffic sign detection CBAM is integrated for enhanced feature extraction along with Ghost Convolutional layers. The result of this study after catering to couple of such challenges such as muddled and erased lanes, low visibility with partially visible road markings etc. can be seen as in Fig. 1 with the red dotted lines indicating the lane detection output after line fitting in the process post processing and green bounding box enclosing the traffic sign detected.

## Method details

Few of the related works on YOLOv8, lane detection and traffic sign detection has been explored to bridge the gap between existing and proposed system such as a study on YOLOv8-ghost-EMA, a lightweight traffic sign detection method that is tailored for embedded devices Luo et al. [1] leverages the Fusion of Efficient Multi-Scale Attention Module with GhostNet backbone, which enables efficient cross-spatial learning achieves 93.5 % mAP on CSUST Chinese Traffic Sign Detection Benchmark (CCTSDB) in poor weather and 82.9 % on Tsinghua-Tencent 100 K (TT100k) datasets, with an inference delay of 0.79 s on Raspberry Pi 4B; it excels previous YOLO versions in speed, memory, and accuracy. However, it lacks detailed implementation and hyperparameter information. Whereas YOLOv8 with EfficientNet as the backbone for real-time road safety alerts Soylu & Soylu [2], utilizes a custom dataset with augmented shadows for enhanced practicality. It achieves exceptional accuracy and speed, handling challenges like shadows, occlusions, and scale variations, with a mAP-50 score of 99.5 % at 640-pixel image size by facing challenges such as acquiring more data, computational resources, and distant sign detection, despite dependency on data quality and hyperparameters.

A work on Convolutional Block Attention Module and Receptive Field Block-You Only Look Once Version 8 (CR-YOLOv8), a model enhancing multi-scale traffic sign detection accuracy using CBAM Zhang et al. [3] addresses challenges in small object detection while maintaining real-time performance with features like Spatial Pyramid Pooling Fusion(SPPF). Despite slight increases in model size, improvements are needed for performance in adverse weather conditions. CBAM is also employed Wang et al. [4] to focus on automatic pavement crack identification using an improved YOLOv8 model to detect cracks quickly and accurately. Utilizing Depth-wise separable convolution and CBAM, the model achieves mAP-50 surpassing a 90 % recognition rate, and F1-score of >97 %. Challenges include the need for significant data and computational resources. YOLOv8-CB introduced for a dense pedestrian detection algorithm tailored for challenging environments like dense crowds and occlusions Liu et al. [5] integrates YOLOv8n and CBAM, showing competitive performance. However, there's room for improvement in detecting severely occluded pedestrians, and the frame rate is comparatively reduced. It sacrifices a bit of detection speed for higher accuracy, aiming to enhance feature representation and localization, resulting in a lighter model with improved accuracy.

For improved traffic sign detection, an enhanced YOLOv8 model has been employed Huang et al. [6] to leverage the TT100 K dataset with data augmentation techniques which aids in small object detection, resulting in a notable increase in mAP by 3.31 % and recall by 3.59 % compared to the base YOLOv8 model. Despite a slightly lower mAP with YOLOv8+DA, the algorithm effectively reduces missed detections and enhances accuracy. A work on road sign recognition system using YOLOv8, focusing on Moroccan road signs with a self-created dataset of twenty classes from various sources Nabou et al. [7] has augmentation with FPN and the model achieves an impressive accuracy of 95 % for both images and videos and has rapid detection and classification capabilities for road signs but lacks an explicit discussion of limitations. Another YOLOv8 experimentation with a benchmark dataset Wang et al. [8] introducing YOLOM, a real-time multi-task framework based on YOLOv8 using the Berkeley Deep Drive Dataset (BDD100 K) dataset with multi-task annotation. It achieves high performance with mAP50 of 81.1 % for object detection and IoU of 28.8 % for lane line segmentation. Despite the lower recall, its lightweight and adaptive nature makes it suitable for various segmentation tasks. A Convolutional Neural Network (CNN-based) combined U-Net and YOLO model variant has been proposed Hotkar et al. [9] employing encoders and decoders for real-time lane and object detection, emphasizing minimal latency and performance by utilizing CULane benchmark and custom datasets. Despite challenges in adverse conditions and complex scenarios, the approach proves computationally efficient and scalable.

The existing research collectively investigates diverse methodologies to advance object detection and recognition. Notable contributions from Luo et al. [1] and Soylu & Soylu [2] focus on real-time traffic sign detection, leveraging techniques like YOLOv8 to achieve remarkable accuracy and speed. The work by Zhang et al. [3] focuses on small target detection and studies by Wang et al. [4] and Liu et al. [5] tackle challenges such as dense crowds and pavement crack identification using refined iterations of the YOLOv8 model, showcasing elevated precision and recall rates. Despite prominent progress, challenges like effectively managing diverse scenarios, managing computational resource demands, and conducting robust analysis exists. Techniques such as data augmentation and lightweight architectures is required to tackle such challenges and Huang et al. [6] and Hotkar et al. [9] has done that; however those experimentations along with Nabou et al. [7] and Wang et al. [8] using benchmark dataset lacks in efficiency and scalability suggesting continued exploration and fine-tuning are essential to support scalability and generalization.

### Proposed System

**Fig. 2:**
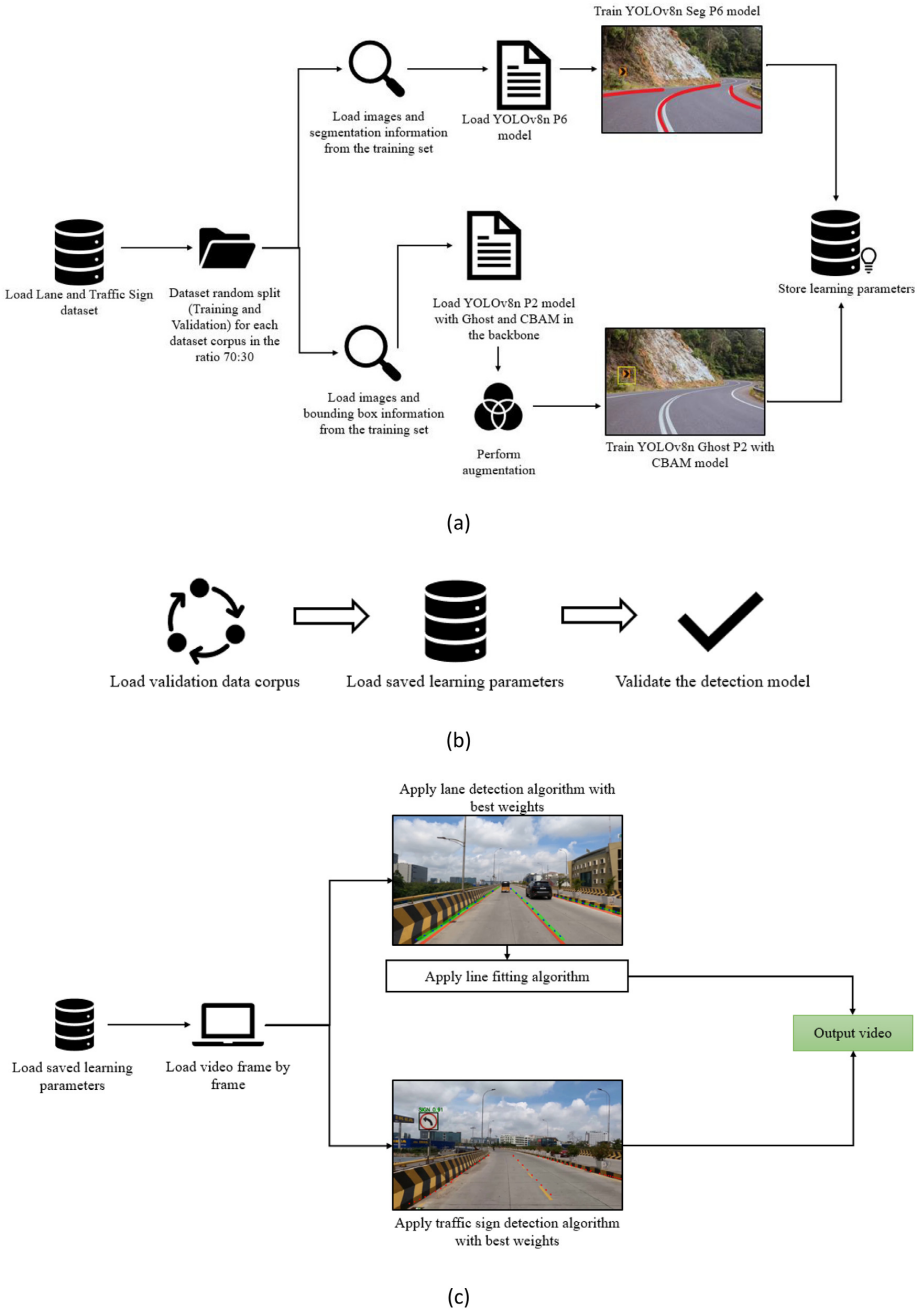
Architecture Diagram of (a) Model Training (b) Model Validation and (c) Model Testing.

Proposed is a Traffic Lane and Sign Detection system which aims towards detecting lanes and signs in a video simultaneously. The proposed flow model is employed by manually scraping data from online sources and preparing it to train and validate the model. Later, the model is tested on videos to detect traffic lanes and signs. Primarily, the proposed system is divided into 3 major phases namely Training, Validation, and Testing. Model training as depicted in Fig. 2(a), comprises both lane and traffic sign dataset which is split into training (70 %), validation (30 %) under every data instance considered to ensure robust evaluation and the lane and sign models are trained individually with their respective datasets. Throughout the training phase, learned parameters are preserved for subsequent stages, ensuring consistency in model performance and the next phase is the validation of the trained models as depicted in Fig. 1(b), which discovers the performance and precision of the proposed system by loading validation data along with the learned parameters and conducting evaluations. Model Testing marks the final phase as shown in Fig. 5(c), where the learned parameters are loaded, and video is processed frame by frame. Lane detection is executed using optimized weights, with the accuracy of detection validated through human interpretation and line-fitting algorithms applied to refine lane definition in the post-processing step of lane detection model. Subsequently, the sign detection model with best weights, completes the comprehensive video analysis.

### Dataset

**Fig. 3:**
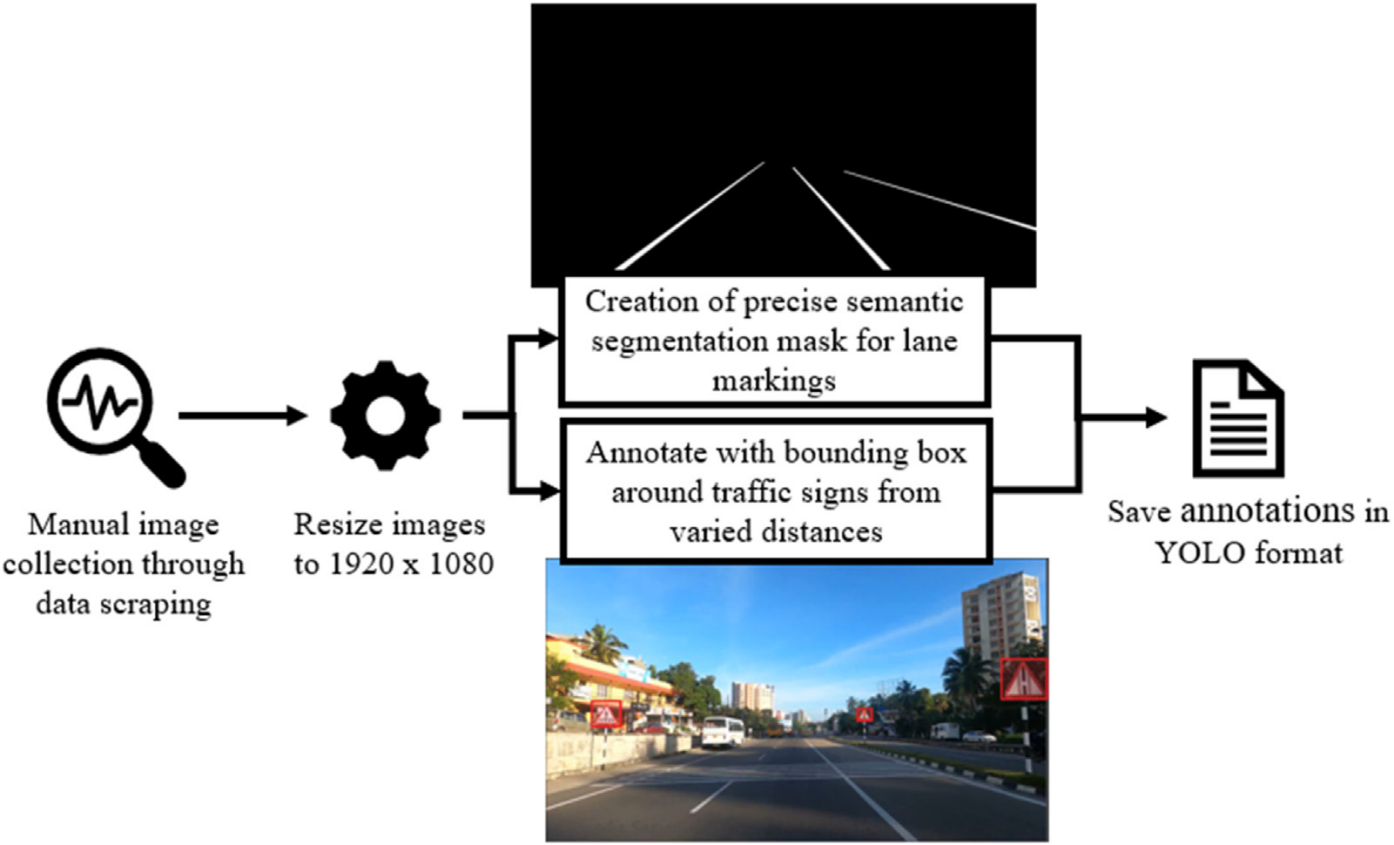
Architecture Diagram of Dataset Collection and Preparation.

The dataset is gathered by manual data scraping on online platforms and the images are preprocessed by resizing the resolution to 1920×1080 pixels. Precise semantic segmentation masks are created for the lane markings, and for traffic sign images, the bounding boxes are annotated for accurate detection. Later, the created annotations are saved in YOLO format and then integrated into the training phase to assist the model in learning as illustrated in Fig. 3

#### Benchmark Datasets

**Fig. 4:**
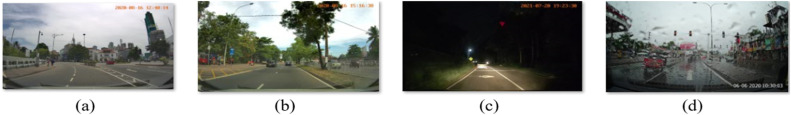
CeyMo Road Marking Dataset Sample for Lane Detection (a) Curve, (b) Day, (c) Night, (d) Rain.

**Fig. 5:**
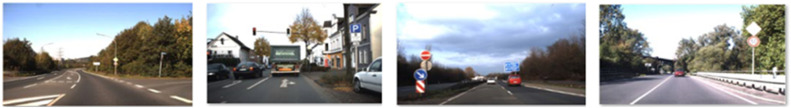
GTSDB Dataset Sample for Sign Detection.

CeyMo Road Marking Dataset has been considered as the Benchmark Dataset for lane detection with 2887 images with a resolution of 1920 × 1080 pixels. It contains a total of 4706 road marking instances in varied challenges like Normal, Crowded, light, Night, Rain, and Shadow in urban, suburban, and rural scenarios as shown in Fig. 4. For sign detection, German Traffic Sign Detection Benchmark (GTSDB) benchmark dataset, consisting of 900 images with varying traffic signs sizes from 16×16 to 128×128, has been used, and the sample is shown in Fig. 5.

#### Custom Dataset

**Fig. 6:**
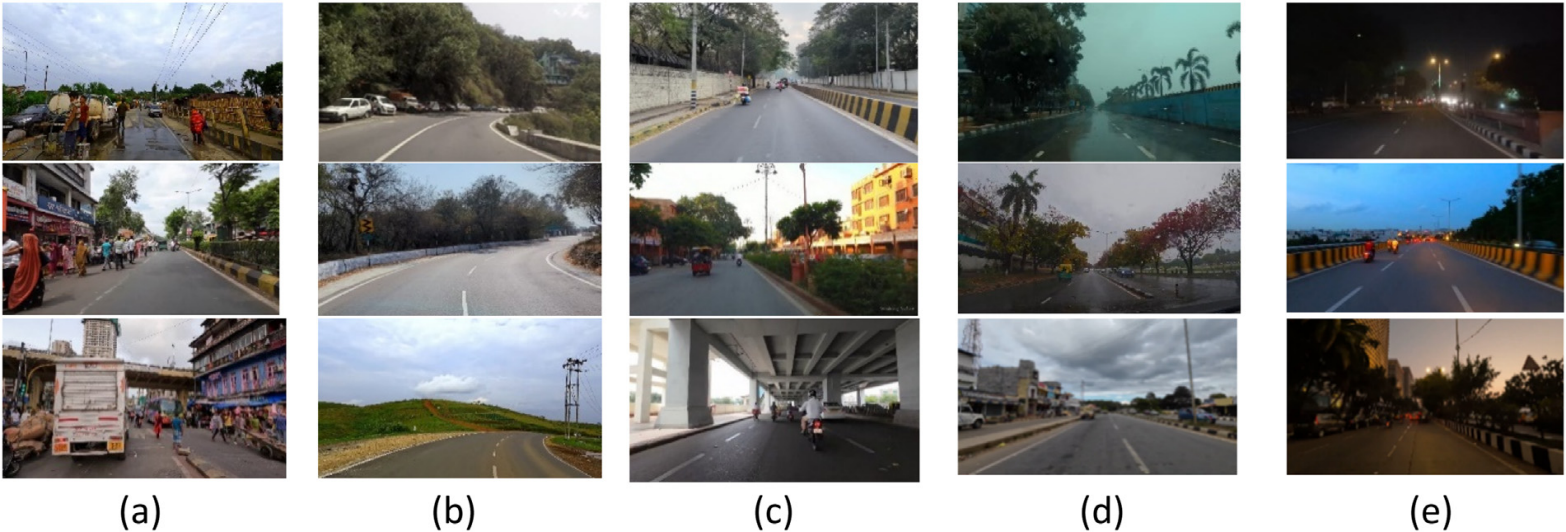
Lane Dataset Sample in Diverse Environmental and Road Conditions (a) Crowd, (b) Curves, (c) Day, Early Morning, (d) Mist, fog, rain, cloudy, (e) Night, sunset.

**Fig. 7:**
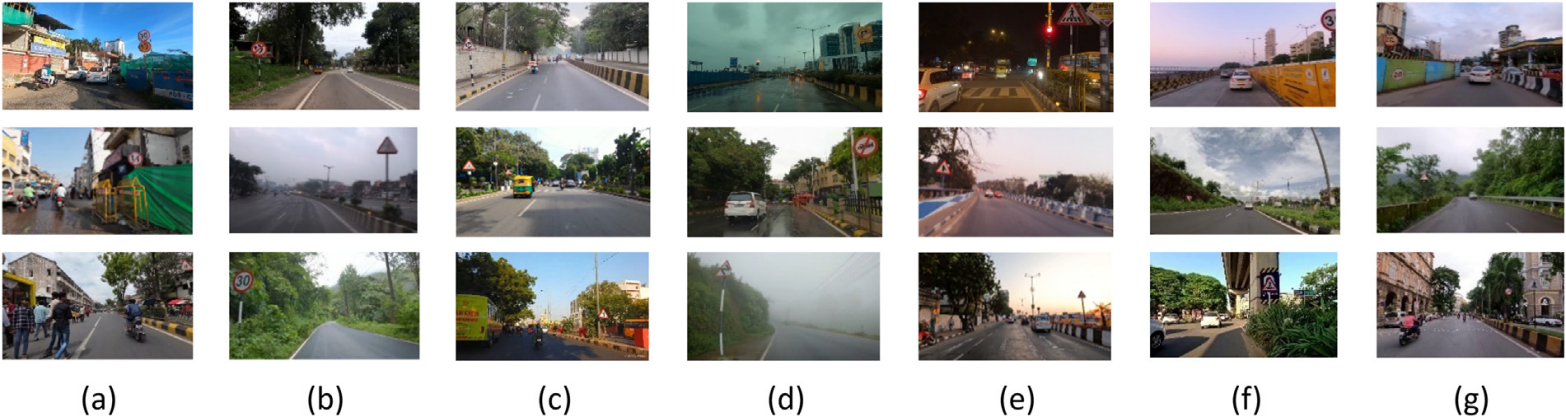
Sign dataset sample in diverse environmental and road conditions (a) Crowd, (b) Curves, (c) Day, Early Morning, (d) Mist, fog, rain, cloudy, (e) Night, sunset, (f) Partially Occluded, (g) Partially Visible.

The lane dataset has mainly five instances such as crowd, curves, day/early morning, mist/fog/rain/cloudy, and night/sunset whereas the sign dataset has two additional instances like partially occluded and partially visible with 2400 images in Indian diverse conditions. Fig. 6 shows the lane dataset sample and Fig. 7 shows the sign dataset sample.

### Proposed Methodology

Leveraging the strength of YOLOv8, adding convolutional blocks for segmentation task and incorporating CBAM for detection to enhance the feature extraction achieving high precision even in complex scenarios like crude weathering and traffic conditions, partially occluded and partially erased lanes, and traffic signs.

#### Lane Segmentation

**Fig. 8:**
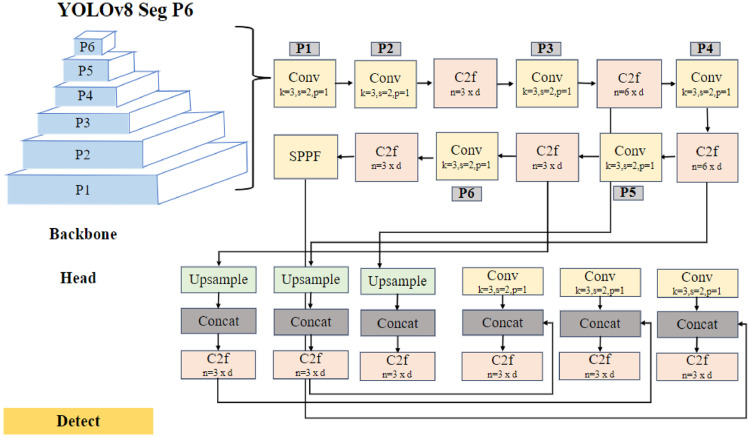
The Architecture of YOLOv8n Segment P6 for Lane Segmentation.

Lane-related images, along with their segmentation information, are fed into the YOLOv8n Seg P6 architecture mentioned in Fig. 8, dedicated to lane segmentation tasks, stands out for its efficient design as it comprises of a six-layer backbone which is P1 to P6 aimed at leveraging its hierarchical feature extraction from the given input images YOLOv8n Seg P6 model for training. Notably, a Spatial Pyramid Pooling Feature (SPPF) block is incorporated post-convolutional layers, enabling multi-scale feature extraction for accurate lane segmentation. It is further strengthened by a C2f (shortcut) module, unique to YOLOv8, which minimizes computational complexity through dense and residual structures. This module facilitates feature transformation and channel adjustments integrating advanced backbone and neck architectures.

In backbone of the architecture, the YOLOv8n model is adjusted with six of the convolutional layers Huijuan et al. [10] to extract and transform input data into meaningful feature representations in segmentation task by applying 3 × 3 kernels to capture spatial details and it YOLOv8n employs Sigmoid Linear Unit (SiLU) Pedro [11] activation function in the CNN block after batch normalization due to its ability to stabilize values independently and to maintain non-monotonic behavior throughout convolution layers. Considering its function of unbounded non-linearity, SiLU is retained in the network for handling complex relationships and gradients crucial for lane and traffic sign detection. It is a product of sigmoidal function as shown below:

$$SiLU\left(x\right)=x\left(\frac{1}{\left(1+e^{-x}\right)}\right)$$

Upsampling in the head of the architecture maintains the aspect ratio of the original image, which is 1920×1080 in the currently used dataset using the nearest neighbor method Madusha [12] and to effectively handle small targets, image size of 1088 is given for model training which scales the images from 1920×1080 to 1088×612. This method ensures that important small target features are preserved for accurate detection. The height h and width w are adjusted i.e., *hr=*h*/612* and *wr=*w*/1088* and calculated using the formula as mentioned below:

$$I_{Uij}=\;I_{yij,xij}$$

Where,

xij=⌈j*wr⌉

yij=⌈i*hr⌉

$I_{Uij}$ upsampled image at *i,j*

Here, *i* and *j* denote the row and column indices of the upsampled image $I_{Uij}$, respectively.

#### Traffic Sign Detection

**Fig. 9:**
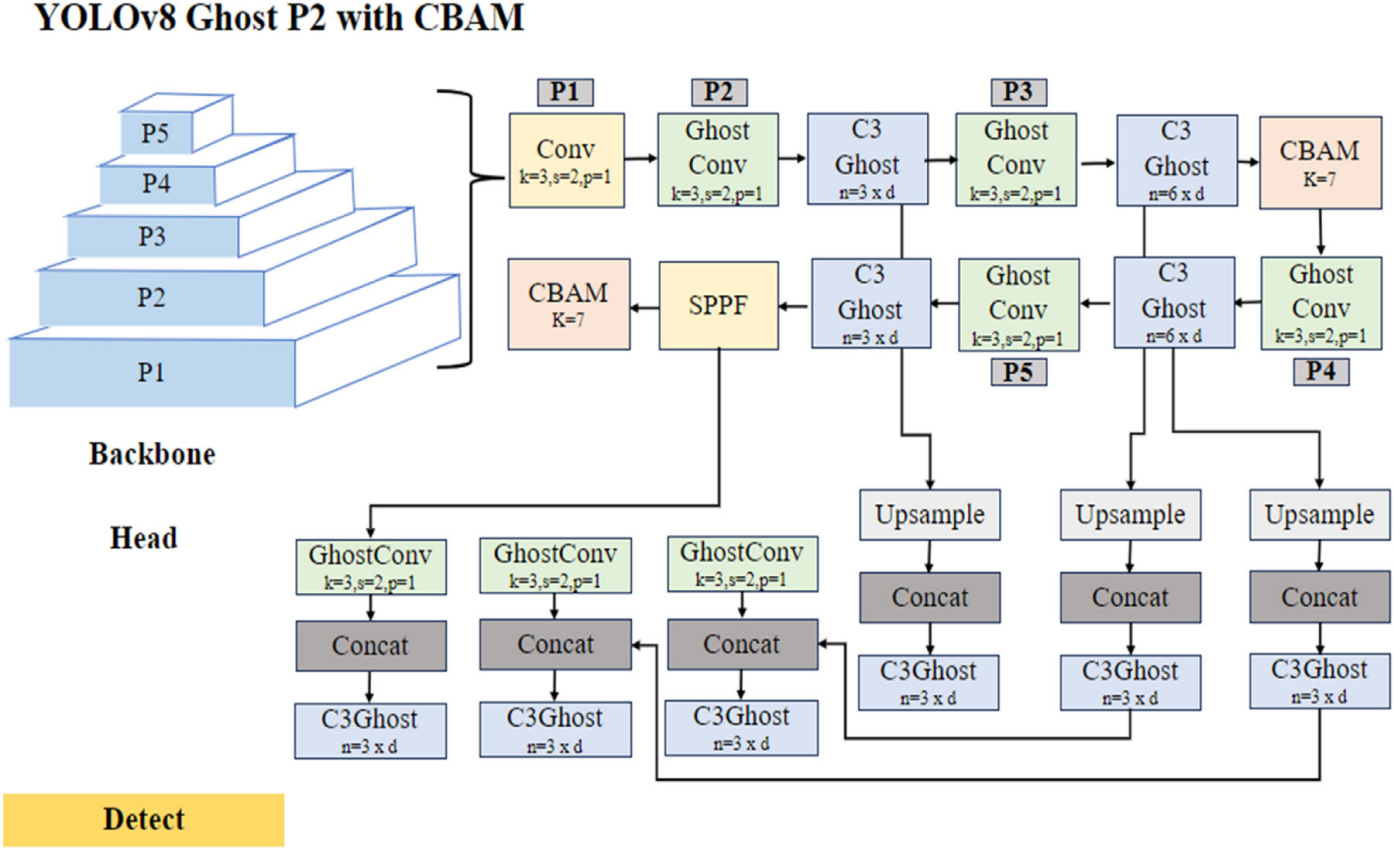
The Architecture of YOLOv8n Ghost P2 with CBAM for Traffic Sign Detection.

The traffic sign data undergoes training on the YOLOv8n P2 model with Ghost and CBAM architecture, enhancing its detection accuracy. The architecture portrayed in Fig. 9, is tailored for traffic sign detection which presents a sophisticated backbone comprising Convolutional, Ghost Convolutional, C3, and CBAM layers, enabling hierarchical feature extraction across different scales. The architecture is augmented with an SPPF layer, filtering feature processing by integrating outputs from P2 and CBAM. The head section incorporates Ghost Convolution layers, Concatenation, and C3Ghost blocks which mainly aids in enhancing the feature representation, along with an Upsample layer for incorporating feature maps, improving spatial resolution for precise object localization that is necessary to locate and localize the traffic signs.

In the sign detection model, Ghost convolution Han et al. [13] is utilized alongside the convolutional layers and upsampling techniques to optimize computational efficiency without sacrificing performance. The usual convolution output is given as shown:

y=x*f+b

Where,

*f*$\in I^{c*k*k*n}$ is the feature map with *n* channels

However, this lightweight variant reduces computational load by employing fewer channels (m ≤ *n*) to generate feature maps to handle small targets using the convolutional operation and it achieves this by linearly combining convolutional operations using a linear operator as mentioned here:

$$y_{g}=x*\;f_{g}$$

Where, $f_{g}\in I^{c*k*k*m}$

$$y_{ij}=LinOp_{i,j\;}\left(y_{gi}\right)$$

Here, *i* = 1, …., m and *j* = 1, …., p and subsequent ReLU activation to introduce non-linearity. Omitting bias terms further updates computation, making Ghost convolution well-suited for scenarios requiring precise yet lightweight convolutional operations, such as in traffic sign detection.

#### Convolutional Block Attention Module (CBAM)

**Fig. 10:**
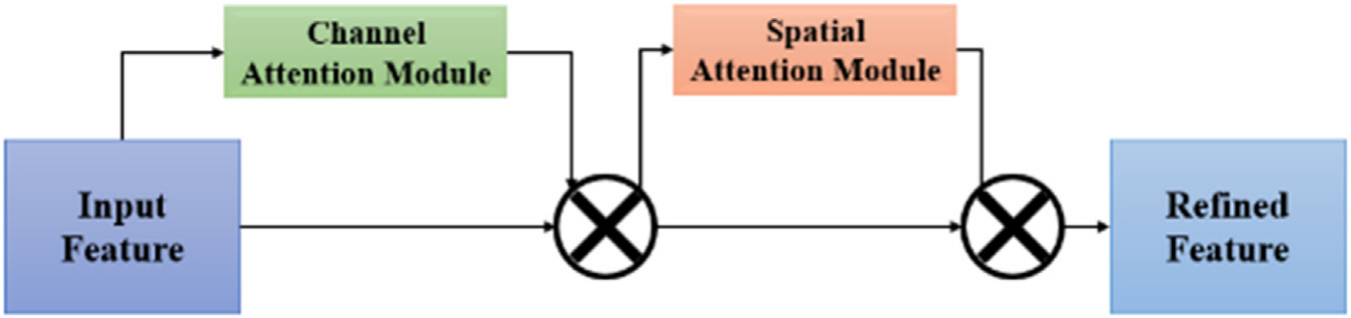
CBAM Mechanism.

CBAM is integrated into the backbone section of the YOLOv8n P2 Sign Detection model to enhance feature extraction as it dynamically enhances feature maps by focusing on relevant regions through channel and spatial attention mechanisms as portrayed in Fig. 10. It helps in extracting deep features including the far, occluded and partially visible signs with the help of Channel Attention that assigns importance weights to informative channels, while spatial attention improves pixel-level localization accuracy to enhance detection by highlighting crucial information and insights. Simultaneously, spatial attention enables the model to concentrate on specific regions of interest.

Given an input feature map I_F_ ∈ I^C * H *^ ^W^, a 1D channel attention map *C_F_*, 2D spatial attention map *S_F_*. The CBAM Woo et al. [14] attention mechanism process’ output refined feature can be outlined as *O_F_* as seen:

$$C_{F}=I^{C*1*1}\left(I_{F}\right)⊗I_{F\;}$$

$$S_{F}=I^{1*H*W}\left(C_{F}\right)⊗C_{F}$$

$$O_{F}=S_{F}$$

Where,

Input feature I_F_ ∈I^C * H * W^

Channel attention map *C_F_*

Spatial attention map *S_F_*

Output refined feature as *O_F_*

#### Data Augmentation

Data Augmentation in sign detection is crucial as techniques like horizontal and vertical flips introduce variations in object orientation, while HSV value adjustments handle changes in lighting and color. Rotation aids in viewing data in different angles, and perspective transformation simulates varying viewpoints. Mosaic augmentation combines images which aid in enhanced learning of the features and mixup generates new examples, improving the traffic sign model generalization ensuring reliable performance in diverse Indian road scenarios for better learning and reduced overfitting risk.

### Method Validation

Tensorflow environment with CUDA integration with Python 3.12 version is employed to carry out the experimentation. Primarily, the experimentation was conducted on 4110 images i.e., 2055 images in each category with real-world Indian driving scenarios covering urban, suburban, and highway conditions from recent years with a resolution of 1920×1080 pixels with an image size of 640 in the batches of 8 over 100 epochs and the experiments were conducted on NVIDIA GeForce GTX 1050 Ti with 8GB RAM. However, to reduce overfitting, the dataset was expanded to 22,400 images and a batch size of 8,16, and 32 were compared based on its performance and the batch size of 16 was used as the standard for this study as it has shown the best result across all the extensive experimentations. The experiments were carried out on NVIDIA RTX A2000 with 12GB RAM GPU after the expansion of dataset and batch size adjustments. To ensure robust model training, each category is divided into training and testing subsets using a 70–30 split, thereby maintaining unbiased representation across all categories.

### Experimentation on initial lane dataset

**Fig. 11:**
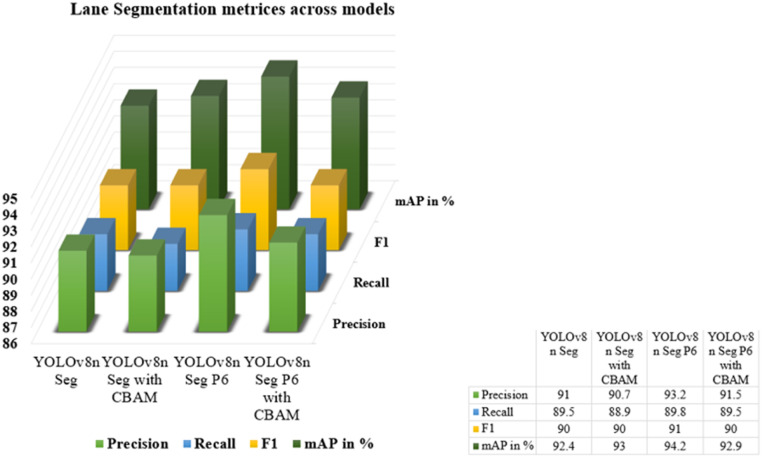
Comparison Graph of Lane Segmentation Metrices Across Models.

YOLOv8n model is added with more convolution layers along with CBAM for experimentation and the results comparison can be seen in Table 1. The YOLOv8n Segmentation model utilizes three layers from P3 to P5 for feature extraction, while incorporating CBAM does not yield significant improvements. In contrast, the YOLOv8n Segmentation P6 model, incorporating four layers i.e., from P3 to P6, achieves a mAP of 94.2 %. Despite CBAM's incorporation to YOLOv8n Seg P6 which increases computational complexity making it a heavy model, the performance is not notably enhanced compared to the former model. The comparison is visually represented in Fig. 11.

**Table 1:** FPS comparison across various YOLOv8n Lane models.

Models	FPS
YOLOv8n Segmentation	108
YOLOv8n Segmentation with CBAM	26
YOLOv8n Segmentation P6	58
YOLOv8n Segmentation P6 with CBAM	53

The YOLOv8n Seg P6 model demonstrates superior performance in lane segmentation tasks, particularly excelling in precision which results in precise lane detection and achieving the highest mAP among compared models represented in Fig. 11.

### Experimentation on expanded lane dataset

The system has been trained using YOLO8n Segmentation P6 model and line fitting algorithm as postprocessing. The Benchmark and custom Datasets were experimented using an image size of 1088 in batch of 16 over 200 epochs and 100 epochs respectively. The experiments on Benchmark dataset resulted in 67.7 % mAP with 56 fps and on the custom dataset, it achieved 90.6 % mAP with 117 fps which is shown in Table 2. An increase in precision and recall on custom dataset signifies improvements in both accuracy and predictions.

**Table 2:** Performance comparison across Benchmark and Custom Lane datasets.

Dataset	Precision in %	Recall in %	F1 in %	mAP50 in %	FPS
Benchmark Dataset	73.9	62.9	68.0	67.7	56
Custom Dataset	89.0	85.5	87.0	90.6	117

**Fig. 12:**
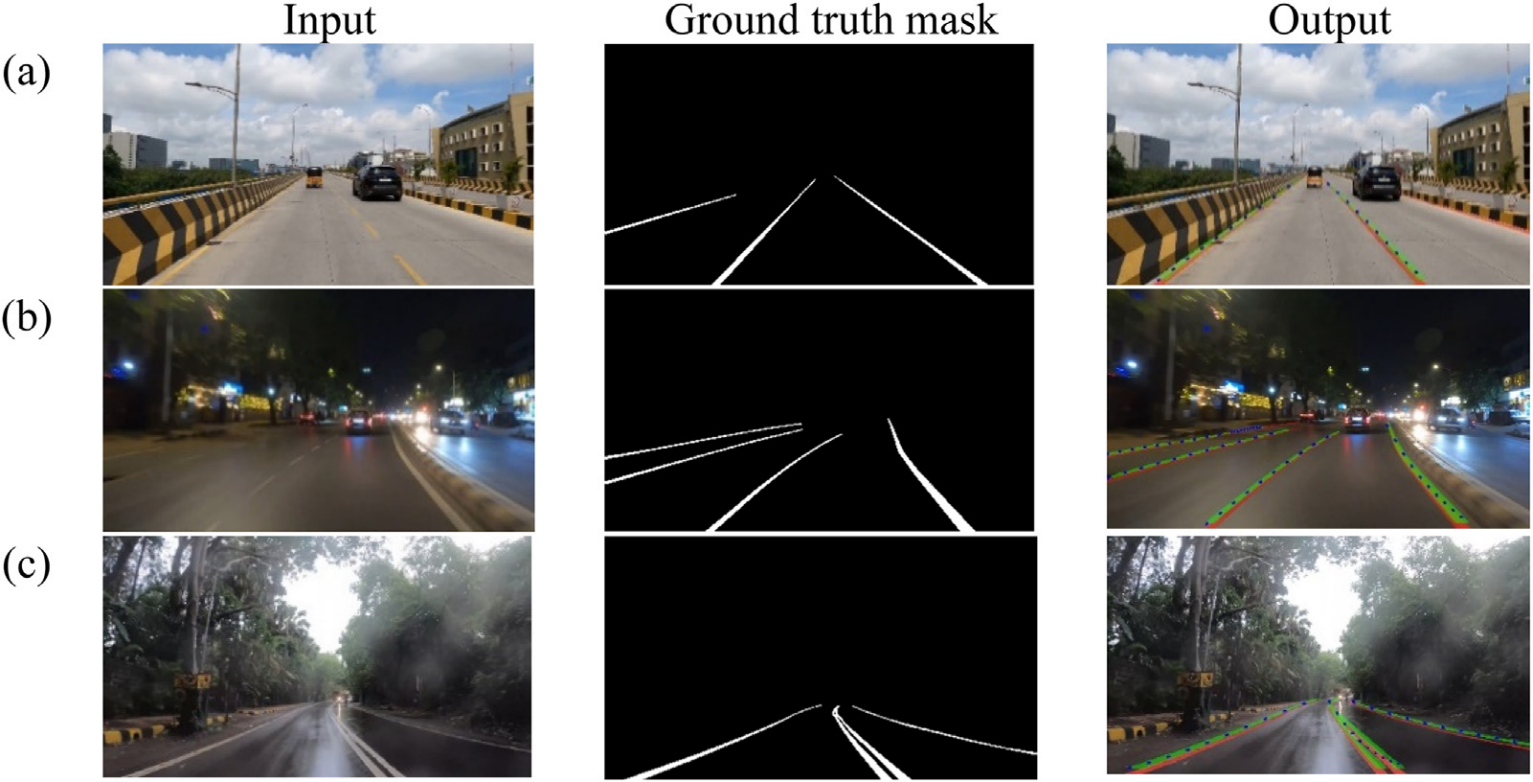
Output of Lane Detection (a) Day, (b) Night, (c) Rain and fog with curves.

Test dataset instances with input images, ground truth masks, and detected lane segmentation output are presented in Fig. 12. Detected lanes are highlighted in green in the output images, a blue line fitting mask from postprocessing and red mask indicated the ground truth. This comparison demonstrates the effective model parameter learning across different instances. As the model does not provide any precision score for the detected lane, Dice Coefficient has been incorporated as part of evaluation metrics.

#### Experimentation on initial sign dataset

YOLOv8n model has been implemented by differing its structure by employing various techniques as given in Table 3 where the model YOLOv8n Detection P2 Ghost and CBAM in backbone and Augmentation has resulted in highest mAP of 84.4 %. The YOLOv8n Detection P2 model employing four layers from P2 to P5 for feature extraction and the YOLOv8n Detection P6 model with Ghost in the backbone, utilizes four layers but demonstrates minimal improvement compared to the Detection P2 model.

**Table 3:** FPS comparison across various YOLOv8n Sign models.

Models	FPS
YOLOv8n Detection P2	147
YOLOv8n Detection P2 Ghost and CBAM in backbone and Augmentation	23
YOLOv8n Detection P6 Ghost in backbone	54

**Fig. 13:**
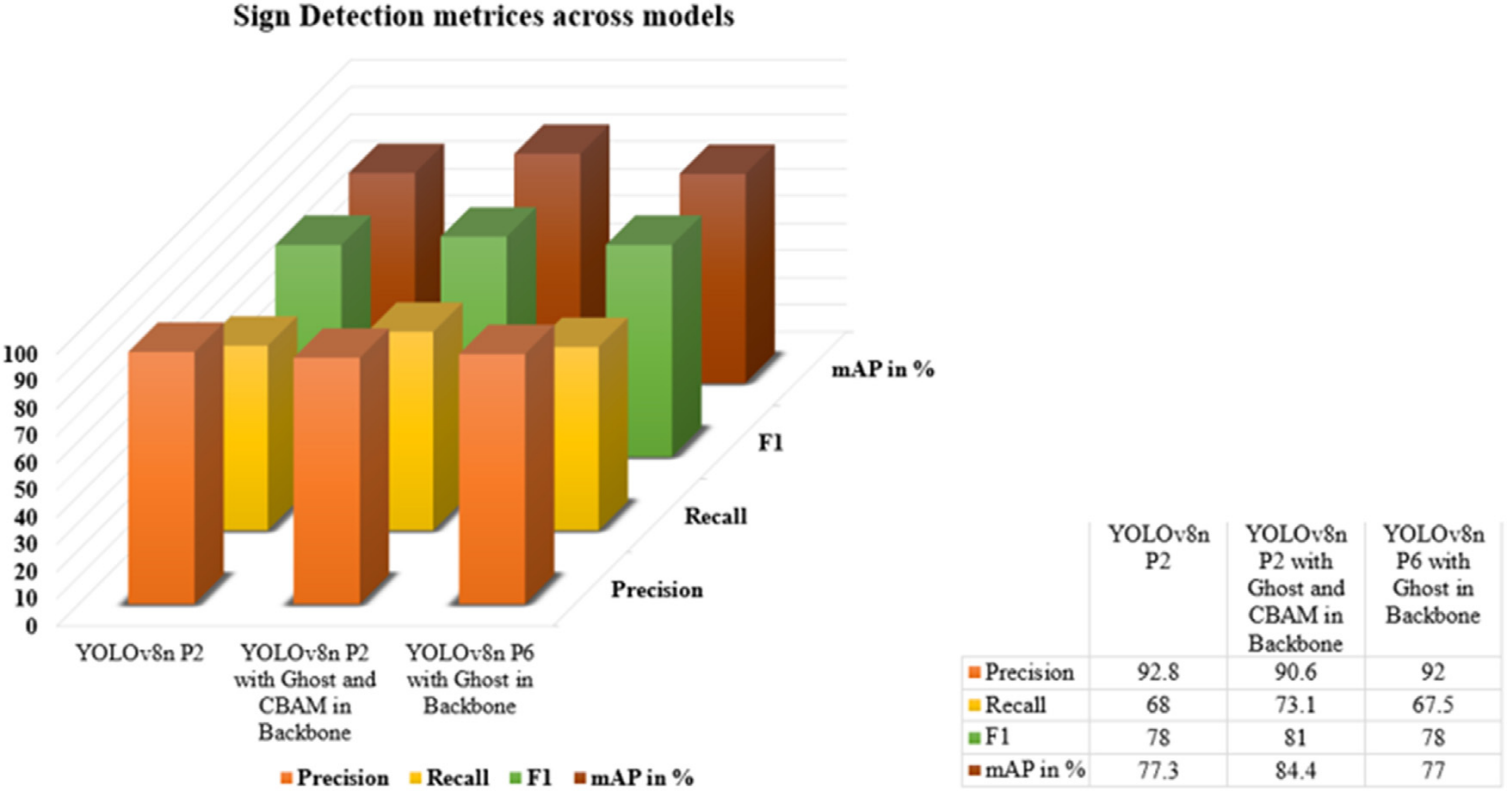
Comparison Graph of Sign Detection Metrices cross Models.

The YOLOv8n P2 model with Ghost and CBAM in the backbone enhanced by data augmentation demonstrated higher performance depicted in Fig. 13, exhibiting the highest mAP, recall and F1 scores, indicating accurate detection and high confidence in predictions. Incorporating Ghost and CBAM into the model led to improvements in mAP and recall, although with a minor tradeoff with respect to precision.

#### Experimentation on expanded sign dataset

The system has been trained using YOLOv8n Detection P2 Ghost and CBAM in backbone and Augmentation model. The Benchmark and custom Datasets were experimented using an image size of 1088 in batch of 16 over 200 epochs and 100 epochs respectively. The experiments on Benchmark dataset resulted in 59 % mAP with 52 fps and on the custom dataset it achieved 81.3 % mAP with 56 fps as shown in Table 4. Introducing Ghost network, CBAM and data augmentation in the architecture significantly improved detection performance with respect to mAP, precision and recall metrices.

**Table 4:** Performance Comparison across Benchmark and Custom Sign Datasets.

Dataset	Precision in %	Recall in %	F1 in %	mAP50 in %	FPS
Benchmark Dataset	79.1	53.8	64.0	59.0	52
Custom Dataset	84.7	71.4	77.0	81.3	56

**Fig. 14:**
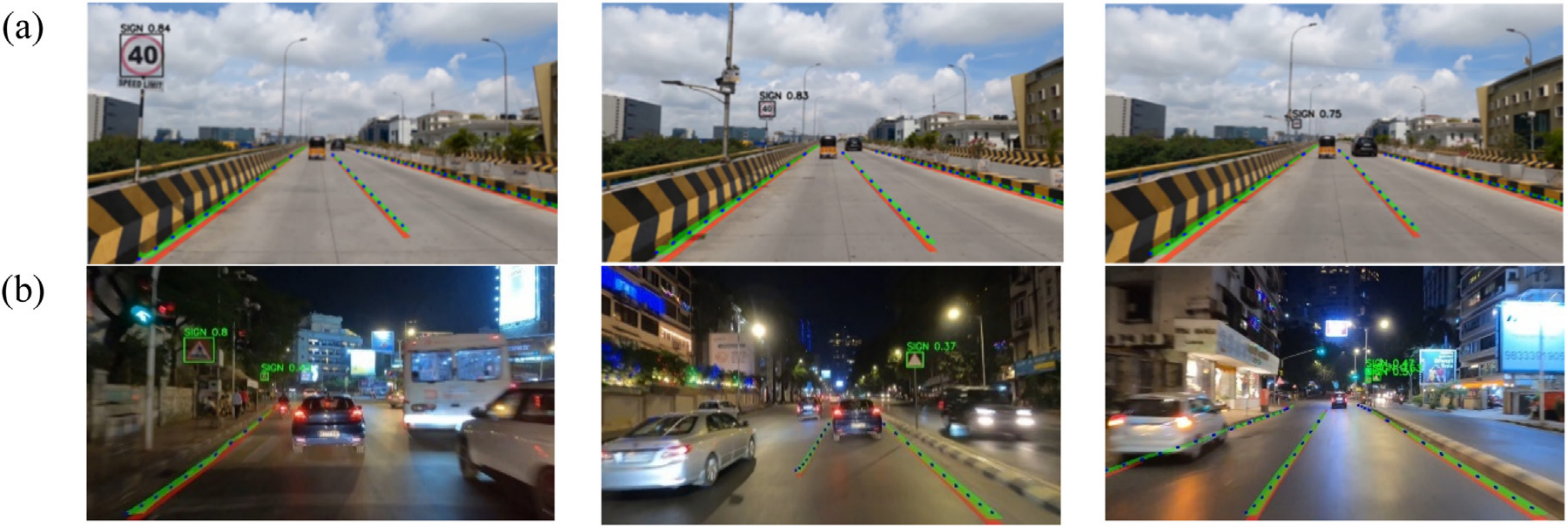
Output of Sign Detection (a) Day, (b) Night.

The sign detection output is given in Fig. 14, where each image includes a bounding box outlining the detected traffic sign, accompanied by a precision value indicating the correctness of the detection whereas an average it is obtained as 80 % precision which is notable.

#### Benchmark dataset vs Indian dataset testing analysis

**Fig. 15:**
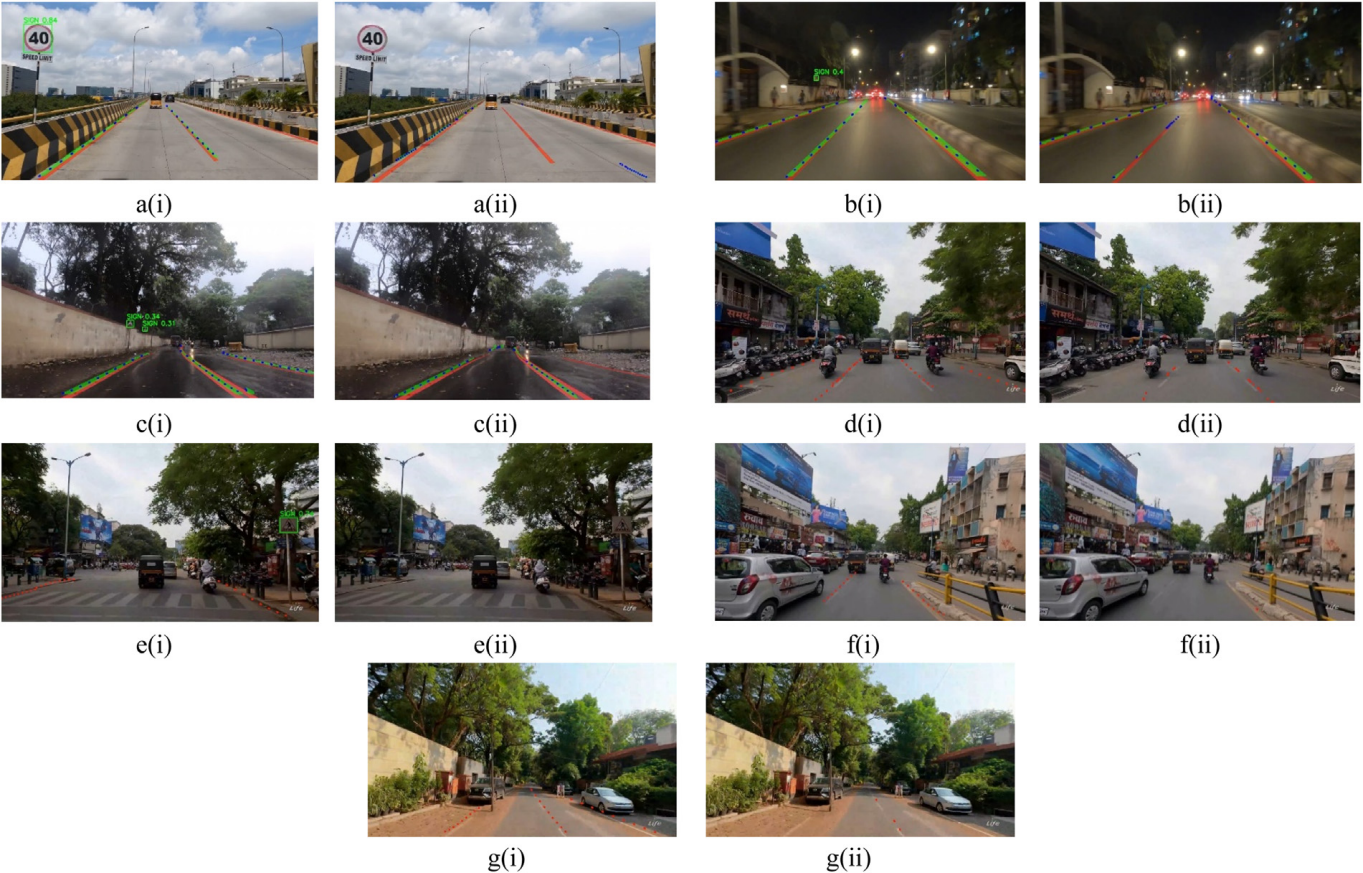
Comparison of Testing Performance on (i) Custom Indian Dataset Trained Model vs. (ii) Benchmark Dataset Trained Model on (a) Day, (b) Night, (c) Mist, Fog, Rain, (d) Crowd, (e) Partially Erased, (f) Partially Visible, and (g) Muddled Lanes.

Performance of the model when trained using a custom Indian dataset and the same when trained using the benchmark dataset is given in Fig. 15 as (i) and (ii), respectively. The comparison focuses on testing scenarios specific to Indian varied road conditions with dotted lines indicating line fitting and green mask is the predicted mask by the proposed model. The test performance of model trained on benchmark dataset reveals limitations in handling the complex Indian urban and suburban scenarios. As illustrated in Fig. 15(a), the sign is partially erased, and the lane markings are feeble. Nonetheless, the model precisely detects them in a(i). In Fig. 15(b), although lane markings and the small traffic sign are present, it is not detected as accurate and precise as in b(i). Indian traffic conditions are often a rush with people, vehicles and with animals positioned haphazardly on the road. Despite these challenges, the model detects the lanes more accurately as seen in Fig. 15d(i). In Fig. 15(e), the sign is partially erased and overshadowed by the trees around making it hard to spot from a distance and the lane markings are feeble. Even so, the model detects them in e(i). The Indian dataset includes scenarios such as rain-soaked roads leading to washed-out lane markings, silt filled roads and small sign boards as depicted in Fig. 15(c), crowded roads with vehicles near lane markings in Fig. 15(f), though the roads are clear from traffic, we can see muddy roads and muddled lanes due to poor maintenance as represented in Fig. 15(g). Regardless of these challenges, the Indian dataset has achieved higher precision in detecting lanes and signs compared to the benchmark dataset.

#### Evaluation metrics

To assess the consistency of the outcomes during the testing phase and to measure the object detection capabilities, the metrices like precision, recall, F1-score, mAP and fps are utilized. Accurate identification of lanes and signs is ensured by precision which measures how the model can label an object, recall gauges how well the model identifies an object whereas F1 score reflects the effectiveness of the model by balancing precision and recall. Mean Average Precision (mAP) provides a detailed evaluation of the model's performance by taking the mean/average of all the average precision. Furthermore, the quantity of the frames per second (FPS) is used to measure how quickly the algorithm detects motion which aids in real-time detection. Here, it means the system processes 1 frame per second at an inference speed of 1000 ms.

$$FPS=\frac{1000}{Inference\;Speed\;\left(milliseconds\right)}$$

In segmentation tasks, the Dice Coefficient metric works in a pixel level as it compares pixels with pixels helping in assessing the segmentation and in detection tasks, the Intersection over Union (IoU) metric determines how much the ground truth box and predicted box overlap. Dice Coefficient was employed for lane detection and it resulted in 0.72 % and IoU employed for sign detection resulted in an impressive score of 91 %.

$$Dice=\frac{2*TP}{\left(TP+FP\right)+\left(TP+FN\right)}$$

$$IoU=\frac{TP}{\left(TP+FP+FN\right)}$$

## Summary

The development of the model for lane detection and traffic sign detection using YOLOv8-based model offers a promising solution for precise detection and tailored to the unique conditions of Indian urban, suburban, and highway environments. Utilization of additional convolutional layers in the head of the architecture addresses the challenge of detecting lanes and signs even when they are partially occluded and partially visible in diverse Indian scenarios. Regardless of the challenges of YOLOv8, the precision obtained for the proposed model is remarkable as the YOLOv8n Seg P6 model for lane detection achieves an impressive training mAP-50 of 90.6 %, and precision inferred by human interpretation is 90.4 % with a Dice Coefficient of 0.72 %. Regarding traffic sign detection, the YOLOv8n P2 model, enhanced with Ghost and CBAM, along with data augmentation techniques that have enhanced confidence in predictions, achieves a training mAP-50 of 81.3 % and a precision of 80 % prediction score on an average given by the model with an IoU of 0.91 %. This study addresses the specific challenges of Indian roads that are not adequately approached by current state-of-the-art models as training the model with benchmark datasets and testing on Indian datasets resulted in suboptimal performance, whereas training the model with Indian datasets and testing on Indian datasets yielded significantly improved results and this highlights the importance of training Indian data for enhancing model accuracy and reliability in diverse real-world scenarios. Though YOLOv8-based approach has concerns about consistency and computational complexities, this approach outperforms alternative models in terms of mAP and F1 scores, showcasing its effectiveness. However, further research is essential to optimize model architectures for improved performance under varying hardware constraints and to enhance the robustness of the system in real-time deployment.

## Limitations

• YOLOv8-based approach has concerns about consistently detecting very small targets and computational complexities such as latency in inference making it less competent for real time deployment.
• Lane detection validation using Dice Coefficient score indicates that there is room for improvement in precise overlapping of the predicted lane mask.
• Sign detection needs intense training to work on blurry and tiny targets for enhanced performance which is dependent on high computational hardware.

## Ethics statements

The dataset was collected from various content creator's videos published on the internet and it has been strictly used for training the lane and sign detection models using YOLOv8 transfer learning approach and there has been no redistribution or publication of the said dataset for monetary purposes. No copyright act has been infringed in the process of utilizing the content creator's work.

## CRediT authorship contribution statement

**H. S. Gowri Yaamini:** Investigation, Methodology, Validation, Writing – original draft. **Swathi K J:** Investigation, Validation, Writing – original draft. **Manohar N:** Supervision, Resources. **Ajay Kumar G:** Conceptualization, Supervision, Writing – review & editing.

## Declaration of competing interest

The authors declare that they have no known competing financial interests or personal relationships that could have appeared to influence the work reported in this paper.

## Data Availability

Data will be made available on request.
